# Abstracts and Selections

**Published:** 1903-06

**Authors:** S. E. Fowler


### Abstracts and Selections.

---

Who of us among the older physicians of today, that can look back to the time when Cod Liver Oil was considered the remedy "par excellence" for all lung afflictions, or diseases of a wasting nature (not the palatable emulsions as we have them today, but the plain, straight oil of the liver of the Norwegian cod, with all its disagreeable odor, taste and nauseating effects) and not say, as has often been said to me by my patients, truly the remedy is worse than the disease. In later years many different forms of emulsions of the oil have been placed before the profession, some of which are fairly palatable preparations and some of which I have used extensively in my practice and in some cases with reasonably good results, but, thanks to scientific research, we *now* have a preparation that, in my opinion, supercedes any of the preparations of Cod Liver Oil, not only as to its palatability, but also as to its physiological action wherever its use is indicated and the large variety of diseases in which it may be used with good effect. I refer to Terraline, which is, strictly speaking, purified petroleum, or in other words, petroleum from which all the objectional qualities have been eliminated, while retaining its full medicinal virtues.

I have used Terraline extensively in the very recent past, and speak from my own observation and knowledge of its curative powers. I have used it in many cases of incipient phthisis, both alone and in combination with beechwood creosote and am well pleased with the results in each case.

I have a case now under my care which I wish to mention. Mrs. O., married, 26 years old, nursing child ten months old. Tubercular diathesis (mother having died with the disease), typical case of phthisis. Had been under care of another physician for six weeks previous to calling on me. Had been taking large doses of Cod Liver Oil until stomach revolted and would retain scarcely anything.

Very weak and emaciated, no appetite, digestion poor. Child very poor, owing to lack of natural nourishment. Former physician had told her to wean child for various reasons. Put her on Terraline with one drop of beechwood creosote to each dose for first six days, then two drops each dose. Vapor bath twice each week, followed by a thorough rubbing of the entire body with Terraline. Has been under my treatment now for two months with wonderful improvement from the first week. Mother rapidly gaining flesh and strength, appetite and digestion good and very little cough, no night sweats and plenty of nourishment for the child, which is now a rosy-cheeked, healthy looking baby. Will be able to report a complete cure in this case in the near future.

This is only one of many cases of phthisis in which I have used Terraline, but I report it to show that Terraline is not only the remedy for the disease proper, but is also a stimulator of nutrition as well as an galactagogue. I have used Terraline in bronchitis, anemia, debility, atonic dyspepsia, neurasthenia, chlorosis, etc., with the *very best* results. In bronchial irritation, croupy coughs and croup it is the remedy "par excellence."

I recently had a case of appendicitis in a boy of thirteen years. Decided not to operate. Impaction in cæcum. No movement for four days. Gave Terraline in maximum doses, more as a lubricant than for any other effect but got good results. Decided to try high enema. Arranged patient so that body would be at an angle of nearly 45 degrees (head downward of course), forced rectal tube well up into transverse colon and injected about a pint of Terraline, made into an emulsion with pure castile soap and water, using about a quart of the preparation and injecting it very slowly, taking about twenty minutes for the injection. Allowed patient to remain in the same position for about fifteen minutes and in about 45 minutes after lowering the bed he had a movement from the bowels of such a nature that it showed that the impaction had been overcome. Continued to give Terraline throughout the further treatment of the case and am fully satisfied that it was a very potent agent in allaying the inflammation and irritation incident to the disease.

Another case under my care at present is that of a young lady of eighteen years. At the time she placed her case in my hands (five months since) she informed me that she did not begin to menstruate until she was past sixteen years old and that her "periods" had always been irregular, very painful and the flow scant.

Very anemic, appetite and digestion poor, some cough of a dry hacking nature, night sweats followed by extreme lassitude. Pale waxy complexion. No history of consumption in family. Put her on Terraline, also vapor baths once each week followed by a thorough rubbing of equal parts of cocoanut oil and Terraline. No other treatment whatever except directions as to diet. I am pleased to state that at the present writing the young lady is practically a picture of health. Weight has increased thirty-two pounds, appetite excellent, digestion perfect, no cough or night sweats, menstruation regular, painless and normal in quantity.

I could report several cases similar to the above if it were necessary to convince the profession of the therapeutical value of Terraline, but will only say in conclusion that if each of the medical fraternity will only test Terraline thoroughly as I have done, they will, I think, say as I do, that in it we have one of the most valuable additions of the age to modern materia-medica.

Respectfully,
S. E. Fowler, M. D.

